# Legacy DDT and its metabolites in Brook Trout from lakes within forested watersheds treated with aerial applications of insecticides

**DOI:** 10.1371/journal.pone.0320665

**Published:** 2025-04-21

**Authors:** Joshua Kurek, Meghan P. Fraser, Bobby J. Nakamoto, Karen A. Kidd, Christopher B. Edge

**Affiliations:** 1 Department of Geography and Environment, Mount Allison University, Sackville, NB, Canada; 2 Department of Biology, Mount Allison University, Sackville, NB, Canada; 3 Department of Biology, University of New Brunswick, Fredericton, NB, Canada; 4 Department of Biology, McMaster University, Hamilton, ON, Canada; 5 Canadian Forest Service, Natural Resources Canada, Fredericton, NB, Canada; University of Tehran, IRAN, ISLAMIC REPUBLIC OF

## Abstract

To manage defoliation from insect outbreaks, about half of the forested land in New Brunswick, Canada, was treated with dichlorodiphenyltrichloroethane (DDT) between 1952 and 1968. Aerial applications of DDT have thus likely increased the risk of chronic effects in aquatic ecosystems from this legacy insecticide given its high persistence in soil and sediments and its bioaccumulation potential within the food web. We investigated DDT and its metabolites (total ΣDDTs =  ∑ DDT +  ∑ DDD +  ∑ DDE) in Brook Trout (*Salvelinus fontinalis*) associated with geospatial data of historical applications to lake watersheds and sedimentary measures of DDT and its metabolites from five “impact” and two “reference” study lakes. Total ΣDDTs in recent lake sediments were significantly correlated with cumulative DDT applied aerially to the lake’s watershed. Brook Trout muscle tissue showed total ΣDDTs that were significantly higher from impact lakes than reference lakes. On average, total ΣDDTs in Brook Trout from impact lakes exceeded ecological guidelines for consumers of aquatic biota by about ten times. Most legacy DDT in Brook Trout and lake sediments were the metabolites ΣDDE and ΣDDD, which suggests the importance of environmental conditions and transport of weathered sources of this organochlorine insecticide to biota. Stable isotopes from fish and common invertebrate prey also suggested that Brook Trout were at a similar trophic position among all study lakes and thus storage pools of legacy DDT likely explain contamination levels within biota. Our findings clearly demonstrate that chronic effects of historical DDT applications likely persist throughout aquatic environments in north-central New Brunswick.

## Introduction

Global production and diversification of synthetic chemicals, such as pesticides, have outpaced well-recognized drivers of global change, such as climate change, eutrophication, and habitat loss, yet synthetic chemicals often remain unstudied in ecological investigations [1]. In the 1950s, the broad-scale use of insecticides became common practice. For example, the forest industry used aerial applications of insecticides to large tracts of land to control pests. If unmanaged, insect pests can cause significant economic losses [2–3]. Between 1952 and 1993 one of the largest aerial spray programs in the world occurred in New Brunswick (NB), Canada, to control outbreaks of eastern spruce budworm (*Choristoneura fumiferana*). During this period, ~ 97% of NB’s 6.2 million ha of forests were treated with at least one application of an insecticide. Early on in this program (1952-1968), ~ 5.7 million kg of dichlorodiphenyltrichloroethane (DDT), a highly persistent insecticide, was applied by airplane to ~ 11.8 million ha of NB’s forests, leaving a legacy of contamination [4–6].

In the environment, DDT degrades into the metabolites dichlorodiphenyldichloroethane (DDD) and dichlorodiphenyldichloroethylene (DDE), with similar toxicological properties as DDT [7–9]. DDT and its metabolites are often well-preserved in deep-water sediments of lakes due to the slow volatilization of these contaminants at low water temperatures and reduced photochemistry [7,10]. A paleolimnological study of five NB lakes within watersheds where DDT was applied showed sedimentary DDTs (∑DDT +  ∑ DDD +  ∑ DDE) dated to the 1970s that were among the highest reported in North America [11]. Peak sedimentary ∑ DDD at Upsalquitch Lake exceeded probable effect levels established by Canadian Council of Ministers of the Environment [7] by ~ 450 times [11]. Today, surface sediments (uppermost 1-3 cm) of NB lakes remain elevated in the toxic metabolites ∑ DDD and ∑ DDE at concentrations [11] that continue to exceed probable effect levels for exposed biota [7]. The high historical inputs of DDT to lake watersheds have resulted in marked compositional shifts of primary consumers, such as crustacean zooplankton, especially at headwater lakes where *Daphnia longispina*-complex were historically abundant in dated sediment cores [11]. These alarming findings prompted questions about the loss of aquatic ecosystem functions and the long-term effects of legacy insecticides in the food web of NB lakes.

Brook Trout (*Salvelinus fontinalis* Mitchell, 1814) are among the top aquatic predators in NB lakes and are often targeted by anglers for consumption. Depending on their age, Brook Trout feed on diverse prey, including zooplankton, aquatic and terrestrial invertebrates, fish, and amphibians [12–14]. Small Brook Trout often feed on sediment-dwelling aquatic invertebrates in the littoral zone of lakes [15]. This reliance on nearshore prey may result in higher exposures and bioaccumulation of DDT in Brook Trout from contaminated lakes given that DDT and its metabolites bind to organic matter in sediments [16]. Benthic invertebrates interact with and live in direct contact with lake sediments and are thus a dietary vector between organic matter-bound contaminants and consumers through bioaccumulation.

Bioaccumulation and biomagnification processes result in higher trophic-level organisms, such as Brook Trout, being vulnerable to chronic exposure to DDT and its metabolites [16,17]. Furthermore, toxic lipophilic organochlorines (i.e., DDT, DDD, and DDE) in muscle tissues of salmonids have been positively correlated with levels observed in their eggs [18], putting offspring at risk. Chronic exposure to DDT and its metabolites negatively affects the central nervous system of fish [19]. DDTs are also endocrine disruptors that may interfere with hormone regulation and lead to reduced fitness of fish [20]. High levels of DDTs in modern fish tissues demonstrate that legacy DDTs can be acquired by aquatic biota [21–24]. Presumably, legacy DDTs are transferred from long-term storage pools in lake sediments and/or watershed soils to biota through dietary routes.

The high persistence of DDT and its metabolites in organic matter-rich lake sediments [11], combined with the widespread application of DDT in NB [6], may have long-lasting impacts on aquatic ecosystems within these watersheds. To address concerns of contamination and ecological impacts to higher trophic-level organisms from legacy insecticides, we studied DDT, DDD, and DDE in Brook Trout from seven remote, dimictic lakes within watersheds across a range of historical pesticide applications that were recently digitized [6]. Our study examines whether contemporary levels of DDTs in Brook Trout reflect historical applications to lake watersheds and whether DDTs observed in this fish species ~ 50 years after aerial application are at levels that pose ecological risks. If so, are factors such as cumulative DDT applied to the watershed or food web structure and/or diet important predictors of DDTs in Brook Trout of NB lakes? Given the elevated DDTs in modern sediments in lakes within sprayed watersheds, we predict that Brook Trout from these lakes will also have high contaminant levels. This retrospective study will help to better understand chronic ecological risks to consumers of aquatic biota associated with persistent organic pollutants, such as DDT and its toxic metabolites DDD and DDE.

## Materials and Methods

### Impact and reference lake study design

**Fig 1:**
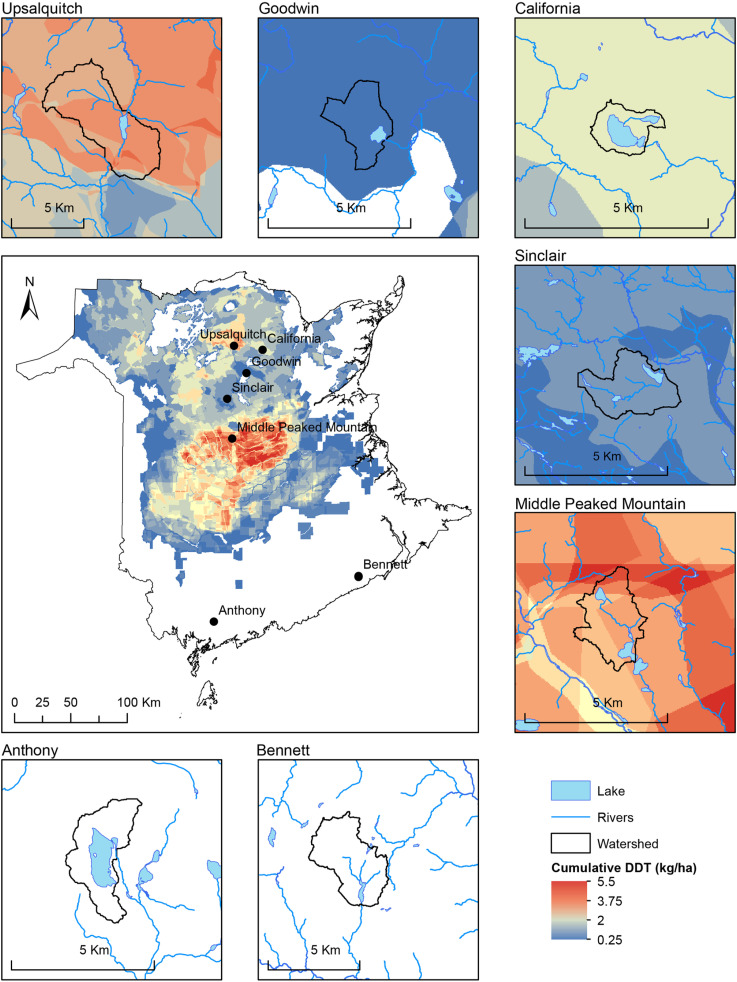
Map of study lakes and cumulative DDT applied between 1952 and 1968 in New Brunswick, Canada. Insets of cumulative DDT applied in watersheds of seven study lakes.

Maps of spruce budworm defoliation and historical DDT applications were used to select five headwater study lakes within watersheds where DDT was applied (i.e., “impact” lakes) between 1952 and 1968. Impact lakes were also chosen to have similar physical characteristics (<70 ha surface area; < 15 m depth) and water quality (Fig. 1; Table 1). Upsalquitch (47.47°N, −66.49°W), California (47.44°N, −66.15°W), Middle Peaked Mountain (46.73°N, −66.51°W), Sinclair (47.05°N, −66.57°W), and Goodwin (47.25°N, −66.35°W) lakes are dimictic, circumneutral, and oligo- to mesotrophic. Forestry activities that include harvesting and road building are the main cause of land-use change within each lake’s watershed in the Atlantic Maritime Ecozone. Conifers such as spruce (*Picea* spp.) and fir (*Abies balsamea*) are the dominant tree species. Mean summer air temperature is 17 °C, mean winter air temperature is -11 °C, and average annual precipitation is about 1000 mm. Two additional headwater systems located in southern NB were chosen as “reference” or control lakes and are located outside of the historical DDT application areas in the province (Fig. 1). The two reference lakes were chosen for their relatively similar fish compositions, physical characteristics, and water quality in comparison to the five impact lakes. Bennett Lake (45.62°N, −65.07°W) is located in Fundy National Park and protected from land-use change associated with forestry activities, whereas forestry activities occur adjacent to Lake Anthony (45.27°N, −66.72°W).

**Table 1:** Physical and chemical characteristics, cumulative DDT application (1952-1968), recent sedimentary total ΣDDTs and metabolites (ΣDDEs and ΣDDDs) from study lakes.

	Anthony (reference)	Bennett (reference)	Goodwin	Sinclair	California	Upsalquitch	Middle Peaked Mountain
Lake surface area (ha)	29.8	31.3	21.4	45.6	45.0	68.1	14.4
Max water depth (m)	12.8	11.1	7.6	8.5	11.5	15.2	9.8
Watershed area (ha)	210.0	1100.0	427.0	1246.0	183.0	3071.0	312.0
Cumulative DDT applied to watershed (kg/ha)	0.0	0.0	0.5	1.1	2.2	3.1	3.2
Total ∑ DDTs (ng/g dwt) in recent sediments	15.5	17.3	40.3	146.5	185.0	123.0	236.9
% of ∑ DDE	48.0	34.0	62.0	59.0	51.0	56.0	51.0
% of ∑ DDD	25.0	43.0	32.0	25.0	44.0	37.0	39.0
% of ∑ DDT	25.0	23.0	5.7	14.0	4.5	6.6	10.0
Total Phosphorus (ug/L)	5.0	6.0	14.0	12.0	22.0	17.0	10.0
Total Organic Carbon (mg/L)	2.8	2.2	2.7	4.4	2.8	2.9	4.4
Colour (TCU)	<5.0^a^	<5.0^a^	8.0	28.0	<5.0^a^	8.0	11.0
Conductivity (µS/cm)	19.0	17.0	37.0	23.0	25.0	67.0	29.0
pH	8.0	7.9	7.5	7.3	7.7	7.5	7.2

^a^Method detection limit was not reached

Surface water samples were collected to describe basic limnological conditions at impact and reference lakes. Goodwin, Sinclair, and Middle Peaked Mountain lakes were sampled on September 2, 2020, California and Upsalquitch lakes on September 10, 2020, Lake Anthony on October 18, 2020, and Bennett Lake on October 28, 2020 (Table 1). Surface water samples were preserved on ice for no more than 48 hours before they were received by the Research & Productivity Council laboratory in Moncton, NB for standard water chemistry measures.

Impact and reference lakes both contain wild populations of Brook Trout and small-bodied fishes common to NB [13]. Middle Peaked Mountain is the only study lake with a history of stocked Brook Trout. The last stocking event occurred in 2000, when ~ 2000 juvenile Brook Trout were added [11]. California, Middle Peaked Mountain, Goodwin, and Bennett lakes are managed wild Brook Trout fisheries with restricted angler access.

### Sediment collection for legacy DDTs

Dated sediment cores from California, Upsalquitch, Sinclair, Goodwin, and Middle Peaked Mountain lakes [11] were used in this study to assess total ∑ DDTs (∑DDT +  ∑ DDD +  ∑ DDE) in the recent sediments of impact lakes. Sediment cores were taken from the deepest basin of each impact lake in 2016. Cores were collected with a gravity corer and sectioned at 0.5-cm intervals [25]. Sediment intervals were then freeze-dried and processed for sedimentary DDT analysis at University of New Brunswick-Saint John following standard methods previously described in detail [11]. Briefly, extraction and clean up followed US EPA protocols 3545A, 3660B, and 3640A. Sediment samples were spiked with a surrogate and extracted with 50:50 dichloromethane: hexane, with extracts concentrated and then run through a gel-permeation chromatography column. Samples were concentrated into hexane and eluted. Fractions were collected from the column and then each was concentrated into isooctane and spiked with pentachloronitrobenzene. Analysis of sedimentary DDTs in each fraction largely followed US EPA protocols 8082 and 8081. Samples were run on a gas chromatography-electron capture detector and quantified using an internal standard calibration. Sedimentary DDTs, specifically, *p,p*’-DDT, *o,p*’-DDT, *p,p*’-DDE, *o,p*’-DDE, *p,p*’-DDD, and *o,p*’-DDD, were confirmed on a second gas chromatography column with greater polarity. We report proportions (%) of the two isomers summed and sedimentary total ΣDDTs as the sum of all six congeners (Table 1). Quality assurance included a method blank, method spike, certified reference material (NIST SRM 1941b Organics in Marine Sediments), and calibration checks.

We used composite intervals that ranged from 1-3 cm of sediment from each impact lake to assess total ∑ DDTs in recent lake sediments (Table 1). Similar to the impact lakes [11], measures of total ∑ DDTs in recent sediments from Anthony and Bennett lakes were obtained from cores collected in the deepest basin in autumn 2020 using a gravity corer [25]. Total ∑ DDTs were measured from composite sedimentary intervals of 0-2 cm (Bennett Lake) and 0-2.5 cm (Lake Anthony) of each undated core from the two reference lakes. Sediments from Lake Anthony and Bennett Lake were analyzed at the Queen’s University Analytical Services Unit following standard methods outlined above [11].

### Sampling of Brook Trout for legacy DDTs

Brook Trout were captured primarily by angling between May 22 and June 12, 2020 at impact lakes and between September 21 and October 2, 2020 at the two reference lakes. Fyke nets were also used as a secondary capture method at Upsalquitch, Bennett, and Anthony lakes. A size range of 20-50 cm was targeted for Brook Trout (n =  5-7 per lake). Trout were sampled and humanely killed following methods that complied with the Canadian Council for Animal Care as approved by Mount Allison University’s Animal Care Committee. A Section 52 licence to fish for scientific purposes was also approved by the Department of Fisheries and Oceans for this study (SG-RHQ-20-004A).

Trout mass and length were recorded before tissue processing. Skin-on fillets of individual fish were freeze-dried and homogenized by mortar and pestle before they were sent for analysis to Queen’s University Analytical Services Unit. All fish tissues at impact and reference lakes were analyzed for organochlorine insecticides by gas chromatography with a mass spectrometer as a detector (GC-MSD) after extraction by Soxhlet extractor and sample cleanup using solid phase extraction chromatography after gel permeation chromatography. Dried fish tissue (2-4 g) was mixed with sodium sulphate and Ottawa sand and spiked with the surrogate decachlorobiphenyl prior to extraction. The samples were Soxhlet extracted with 250 mL of dichloromethane for 6 hours (~2-3 cycles per hour). Negative control samples were blank extracts that received only decachlorobiphenyl, Ottawa sand, and sodium sulphate. Positive control samples were blank samples spiked with a chlorinated pesticide mixture. Tissue samples were concentrated and cleaned up and fractionated with gel permeation chromatography into a lipid fraction and a pesticide fraction. The pesticide fraction was twice concentrated, and the first fraction eluted on a Florisil column with hexane. This fraction was then concentrated 5-fold, then 100 µ L was transferred to a GC vial and an internal standard added to each vial. In this analysis, *p, p*′ -DDT, *o, p*′ - DDT, *p, p*′-DDE, *o, p*′-DDE, *p, p*’-DDD, and *o, p*′-DDD were measured. Therefore, we refer to ΣDDT, ΣDDE, and ΣDDD as the sum of their two respective isomers. Total ΣDDTs are the sum of all six congeners (Table 2). Control samples, replicates, and blanks were completed for 10% of the fish tissue samples. Method detection limits for each isomer were 10 ng/g dry weight. Isomers of DDTs were reported as ng/g dry weight (ppb). The lipid fraction of fish tissue was analyzed gravimetrically and reported as percent dry weight (Table 2).

**Table 2:** Mean (±SD) measures of skin-on Brook Trout muscle tissue from study lakes. When samples were below minimum detection limits of DDTs, DDEs, and DDDs measures were converted to half the method detection limit of 10 ng/g dry weight.

	Anthony (reference)	Bennett (reference)	Goodwin	Sinclair	California	Upsalquitch	Middle Peaked Mountain
*n*	6.0	7.0	6.0	6.0	6.0	6.0	5.0
Lipid (%)	12.4 ± 2.1	4.2 ± 3.0	7.4 ± 2.8	7.2 ± 1.2	11.1 ± 3.6	10.2 ± 3.9	13.6 ± 1.9
Mass (g)	162.0 ± 43.0	109.0 ± 17.0	308.0 ± 172.0	308.0 ± 86.0	310.0 ± 64.0	180.0 ± 102.0	502.0 ± 186.0
Length (cm)	23.0 ± 2.2	22.3 ± 1.2	31.5 ± 3.5	32.1 ± 2.6	32.1 ± 1.4	27.0 ± 3.9	34.8 ± 4.2
∑DDE in tissue (ng/g dwt)	15 ± 4	a	58.0 ± 35.0	642.0 ± 130.0	624.0 ± 194.0	650.0 ± 130.0	394.0 ± 109.0
∑DDD in tissue (ng/g dwt)	a	a	11.0 ± 2.0	103.0 ± 44.0	241.0 ± 112.0	73.0 ± 59.0	108.0 ± 39.0
∑DDT in tissue (ng/g dwt)	a	a	a	67.0 ± 36.0	33.0 ± 14.0	19.0 ± 9.0	a
Total ∑ DDTs in tissue (ng/g dwt)	35.0 ± 4.0	30.0	79.0 ± 35.0	813.0 ± 190.0	899.0 ± 298.0	742.0 ± 411.0	511.0 ± 144.0
Wet weight equivalents of Total ∑ DDTs in tissue (ng/g)	7.0^b^	6.0^b^	15.0^b^	162.0^b^	179.0^b^	148.0^b^	102.0^b^

^a^Denotes that all measures were below the method detection limit.

^b^Dry weight total ∑ DDTs were converted to wet weight equivalents with an assumption of 80% moisture content in the fish tissue [26].

### Stable isotopes of biota

Benthic invertebrates were sampled from the littoral zone of impact and reference lakes between July 15 and 22, 2021 with 400 µm kick-nets at ~ 1 m water depth near where angling occurred. Samples were live sorted using three sieves (4.75 mm, 1.65 mm, and 500 µm), and grouped into taxonomic units. Chironomidae larvae were sampled from the profundal zone with a Ponar sampler near to where sediment coring occurred. Invertebrates were preserved in 95% ethanol and identified to family level using a stereoscope. Zooplankton were also sampled from the epilimnion with an 80-µm net from the center of each lake with a horizontal tow of approximately 15 m. Only one homogenized zooplankton sample was collected from each lake.

Prior to stable isotope analysis, benthic invertebrates, zooplankton, and skin-on Brook Trout fillets were freeze-dried and homogenized with a mortar and pestle. For analyses, approximately 2 mg of sample was weighed in a tin capsule. Whereas one composite zooplankton sample was analyzed from each lake, closely related benthic macroinvertebrates were pooled for isotope analysis. Samples were analyzed for δ^13^C and δ^15^N in continuous-flow mode at Mount Allison University using an Elementar PyroCube Elemental Analyzer and Isoprime Precision Isotope Ratio Mass Spectrometer. All δ^13^C and δ^15^N are reported in standard delta notation relative to the primary reference materials Vienna Peedee Belemnite and atmospheric N_2_, respectively. Raw isotope measurements were normalized to international scales using replicate measurements of three certified USGS reference materials interspersed among unknown sample analyses: USGS61, n =  14: δ^13^C =  −35.05 ±  0.04 ‰; δ^15^N =  -2.87 ±  0.04 ‰, USGS62, n =  12: δ^13^C =  -14.79 ±  0.04 ‰; δ^15^N =  20.17 ±  0.06 ‰, and USGS63, n =  4: δ^13^C =  −1.17 ±  0.04 ‰; δ^15^N =  37.83 ±  0.06 ‰. We also analyzed certified (USGS40, n =  12: δ^13^C =  −26.39 ±  0.04 ‰; δ^15^N =  -4.52 ±  0.06 ‰, USGS41a, n =  10: δ^13^C =  36.55 ±  0.08 ‰; δ^15^N =  47.55 ±  0.06 ‰, USGS77, n =  6: δ^13^C =  -30.71 ±  0.07 ‰) and in-house (Ammonium Sulfate, n =  6: δ^15^N =  −0.75 ±  0.10 ‰, L-Glutamic Acid, n =  20: δ^13^C =  -16.40 ±  0.07 ‰; δ^15^N =  -6.76 ±  0.1 ‰) standard materials which were not used in the normalization procedure to constrain measurement bias. Lastly, 13 isotope samples were run in duplicate with a mean disagreement of 0.11 ±  0.08 among replicates. Using standard methods [27] we then determined analytical uncertainties of 0.20 ‰ and 0.28 ‰ for δ^13^C and δ^15^N, respectively.

### Statistical analyses

**Fig 2:**
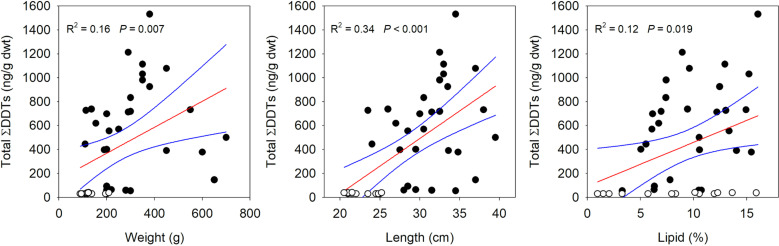
Total∑ DDTs (ng/g dry weight) in Brook Trout muscle tissue compared to weight (A), length (B), and percent lipid (C). Fish from reference lakes (open circle, n = 13 trout) and lakes from watersheds treated with DDT (closed circle, n = 29 trout) are denoted.

Statistical analyses were conducted in R version 4.3.2 [28]. Isomers of ΣDDT, ΣDDD and ΣDDE below minimum detection limits in Brook Trout tissue were recorded as half the detection limit. Linear regression was used to assess relationships between total ΣDDTs (∑DDT +  ∑ DDD +  ∑ DDE) within Brook Trout muscle tissue and fish weight, length, and percent lipid (Fig. 2). In addition, Pearson correlation was used to test the linear relationship between a lake’s cumulative DDT applied to the watershed and total ΣDDTs in recent sediments (Table 1). Both measures from the seven study lakes passed the Shapiro-Wilk test for normality. For all statistical tests significance was accepted at <  0.05.

We also tested if total ΣDDTs in Brook Trout differed between reference and impact lakes using a nested analysis of variance (ANOVA) with a linear mixed-effects model [29]. Historical application of DDT to a lake’s watershed was recognized as fixed (two levels: reference or impact). Lake as a random factor (two reference and five impact lakes) was nested within the fixed factor. Assumptions of ANOVA were assessed with a Levene’s test for homogeneity of variance and a Shapiro-Wilk test for normality. Total ΣDDTs from Brook Trout muscle tissue were then transformed with a natural logarithm to improve homogeneity of variance and normality, although following transformation data were not normal due to bimodality (W =  0.82, *p* <  0.001). We predicted that Brook Trout from impact lakes would contain higher total ΣDDTs in their muscle tissue.

Stable isotope data were used to estimate Brook Trout trophic position and resource use with the tRophicPosition package [30]. Our model framework used uniform priors and assumed that Brook Trout acquired organic matter from two distinct pathways: profundal-pelagic (i.e., open water) and littoral (i.e., littoral zone, nearshore). Each lake’s littoral end-member was composed of benthic macroinvertebrates collected at <  1 m depth near the lake shore. Conversely, the open water end-member was composed of profundal zone invertebrates and pelagic zooplankton. We used typical values [31] for diet-tissue offsets: δ^13^C =  0.39 ±  1.3 ‰; δ^15^N =  3.4 ±  0.98 ‰. A separate model was run for each lake and each model was composed of five parallel chains with 10,000 iterations burn-in followed by 10,000 adaptive iterations and 100,000 posterior sampling iterations. In all cases, Gelman-Rubin convergence diagnostics were near one ( < 1.1). Model outputs are extracted and presented as posterior distribution modes and 95% credible intervals.

## Results

### DDT application to watersheds and recent sedimentary concentrations

The cumulative DDT applied between 1952 and 1968 was generally highest in central NB within the Miramichi River watershed, but also high within the headwaters of the Upsalquitch River watershed (Fig. 1). Of the five impact lakes within DDT-applied watersheds, cumulative DDT was greatest within Middle Peaked Mountain and Upsalquitch watersheds at >  3 kg/ha (Table 1). The Goodwin watershed had the lowest cumulative DDT applied of the five impact lakes at ~ 0.5 kg/ha. The number of applications of DDT also varied across years between watersheds. Middle Peaked Mountain and Upsalquitch watersheds were treated for eight and seven years, respectively. Sinclair and Goodwin watersheds were treated for two and one years, respectively.

Total ΣDDTs in recent sediments from the seven lakes were strongly correlated with cumulative DDT applied to the watershed (*r* = .86, *p* =  0.012). On average, metabolites ΣDDE and ΣDDD comprised ~ 56% and 35%, respectively, of the sedimentary concentrations from impact lakes, which suggests no recent DDT inputs and highlights the importance of weathered sources of legacy DDT. Reference lakes Anthony and Bennett had no DDT applied directly to their watersheds, which was reflected in the lowest sedimentary total ΣDDTs at <  20 ng/g, about half of the concentration observed at the impact lake (Goodwin) with the lowest cumulative DDT applied to the watershed (Table 1). On average, the reference lakes showed metabolites ΣDDE and ΣDDD at 41% and 34%, respectively, of sedimentary total ΣDDTs.

### ΣDDTs, ΣDDEs, and ΣDDDs in Brook Trout

The concentrations of ΣDDT, ΣDDE, and ΣDDD in muscle tissue of Brook Trout from the two reference lakes were below the method detection limit in 9 of 13 fish (Table 2). Only four Brook Trout at Anthony showed ΣDDE above method detection limits and reflected the dominance of ΣDDE in the recent sediments of this lake. When converted to wet weight equivalents, total ΣDDTs in Brook Trout at the reference lakes (Anthony and Bennett) were below the CCME guideline for the protection of wildlife consumers of aquatic biota of 14 ng/g [8].

Of the five impact lakes from watersheds where DDTs were applied, ΣDDT and ΣDDD were observed below method detection limits in 14 of 29 fish and 5 of 29 fish, respectively (Table 2). Overall, California recorded the highest average total ΣDDTs at 899 ng/g dry weight and all fish from California showed concentrations of ΣDDT, ΣDDE, and ΣDDD above method detection limits. Brook Trout from Goodwin showed the lowest average total ΣDDTs compared to other impact lakes at 79 ng/g dry weight (Table 2). On average, ΣDDE comprised ~ 76% of the total ΣDDTs from Brook Trout at the impact lakes and reflected the high % ΣDDE in the sediments. Following conversion to wet weight equivalents, mean total ΣDDTs in Brook Trout at impact lakes ranged from 15 ng/g at Goodwin Lake to 179 ng/g at California Lake (Table 2), all above the CCME guideline of 14 ng/g wet weight [8] (Table 2). Brook Trout from these five impact lakes showed total ΣDDTs that were on average about ten times greater than the CCME guideline [8].

**Fig 3:**
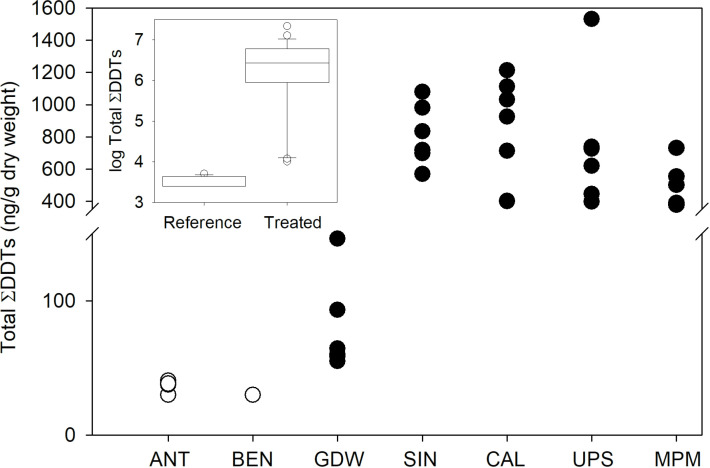
Total∑ DDTs (ng/g dry weight) in Brook Trout muscle tissue from two reference lakes (open circle, n = 13 trout) and five lakes (closed circle, n = 29 trout) from watersheds treated with DDT. Inset shows transformed measures used in statistical analysis.

Log transformed total ΣDDTs from Brook Trout at all seven study lakes were modelled as the linear sum of the y-intercept (3.49 ± 0.64 SE) and DDT application (2.60 ± 0.76 SE), with a statistically significant net effect of DDT application to a lake’s watershed. Total ΣDDTs in Brook Trout muscle tissue were significantly higher from impact lakes than reference lakes (*F*_*1,5*_ =  11.68, *p* =  0.018) (Fig. 3). The fixed effect of DDT treatment to a lake’s watershed accounted for 62.0% of the variation in transformed total ΣDDTs in Brook Trout muscle tissue. Lake as a random factor nested within DDT treatment was also a significant term (*p* <  0.001).

Additionally, there were positive relationships between total ΣDDTs in Brook Trout and fish weight (R^2^ =  0.16, *p* =  0.007), length (R^2^ =  0.34, *p* <  0.001), and percent lipid (R^2^ =  0.12, *p* =  0.019) across all seven study lakes (Fig. 2). However, total ΣDDTs within the 13 Brook Trout from the two reference lakes had a smaller range of sizes than the impact lakes and showed no relationships with weight, length, or lipid percent.

### Stable isotope analysis

**Fig 4:**
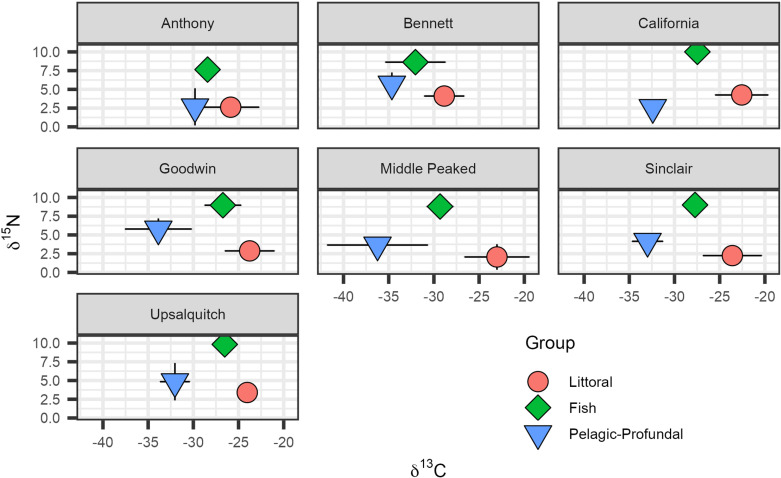
Carbon and nitrogen stable isotope composition ( ± 1 SD) of littoral macroinvertebrates (n = 5-7/lake), Brook Trout muscle tissue (n = 5-7/lake), and composite profundal Chironomidae and pelagic zooplankton samples (n = 2/lake).

**Fig 5:**
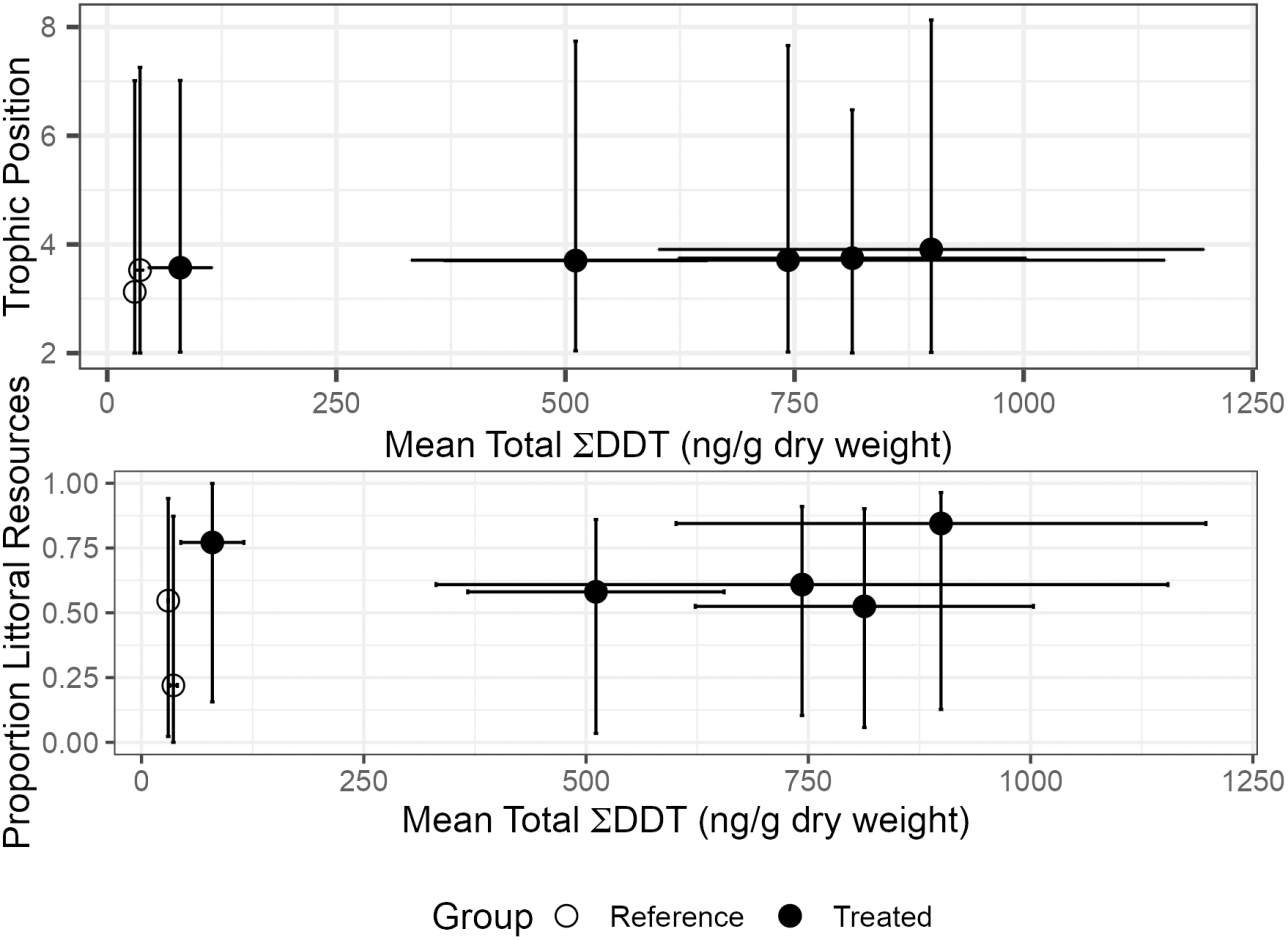
Model estimates for trophic position and resource use of Brook Trout compared to mean Total∑ DDTs (ng/g dry weight) of Brook Trout. Standard deviations are plotted for Total ∑ DDTs and 95% credible intervals are used for littoral resource use and trophic position. Open circles denote two reference lakes and closed circles denote five impact lakes.

Brook Trout stable isotope compositions were similar among all study lakes. Overall δ^13^C and δ^15^N means were −28.4 ±  2.5‰ and 9.0 ±  0.9‰, respectively (n = 42). Moreover, relationships among prey group stable isotope values and those measured in Brook Trout were similar between lakes. For example, although pelagic-profundal and littoral prey both had δ^15^N which was relatively lower than was measured in Brook Trout, pelagic-profundal prey items had more negative δ^13^C than those from littoral areas (Fig. 4). Overall, mode estimates of trophic position of Brook Trout were similar across all study lakes and ranged between 3.2 and 3.9 in Bennett and California lakes, respectively, whereas mode estimates for littoral resource use ranged from 0.23 in Anthony Lake to 0.86 in California Lake (Fig. 5). Nonetheless, there was substantial overlap among lakes of 95% credible intervals for most stable isotope-derived model estimates.

## Discussion

We investigated legacy DDTs in Brook Trout and modern lake sediments in relation to recently digitized geospatial data [6] of cumulative DDTs applied to lake watersheds between 1952 and 1968. Notably, our findings show that Brook Trout in remote lakes from north-central NB are currently exposed to, and contain in their muscle tissue, high levels of total ∑ DDTs that exceed ecological guidelines [8] by an average of ten times. Historical applications of the insecticide DDT by airplane to the watershed’s forests during the 1950s-1960s are the main cause of legacy DDT pollution, which differs largely from other commonly reported input pathways of DDT and its metabolites to the aquatic environment, such as direct industrial discharge [32], glacial meltwater [33], atmospheric long-range transport [34–36], and localized agricultural or urban activities [37–39]. We estimate that of NB’s ~ 2500 lakes, about 500 lakes greater than 3 ha in surface area are located within watersheds where DDT was applied, often at relatively high concentrations and over multiple years. Due to the persistence of DDT, DDD, and/or DDE in profundal lake sediments at elevated concentrations, biota may be at risk of exposure to high levels of DDT, DDD, and/or DDE through diet or habitat use across many NB lakes where DDT was applied to the watershed by airplane. Exposure via sedimentary storage pools of persistent organic pollutants is recognized as a major contaminant pathway to biota [32,35]. If our findings from five impact lakes are scalable, we estimate that approximately 25% of lakes in NB may record concentrations of legacy DDTs in predatory fish and surface sediments that exceed ecological guidelines [7,8]. However, a complex relationship likely exists between cumulative DDTs applied to a lake’s watershed and total ΣDDTs in the aquatic ecosystem that warrants further investigation.

### Legacy DDTs and its metabolites in NB lakes

We expected a positive relationship between total ΣDDTs in recent lake sediments and Brook Trout muscle tissue due to the cumulative DDT applied to these lake watersheds and its environmental persistence and potential to bioaccumulate and biomagnify. Indeed, our findings show that sedimentary total ΣDDTs are a useful predictor of total ΣDDTs in Brook Trout muscle tissue. Given these results, lake sediments appear to be a major source of legacy DDTs [11], including DDDs and DDEs, to the food web of hundreds of NB lakes located in watersheds treated with DDTs [6]. Our results from Anthony and Bennett lakes also show that global transport and deposition of DDTs [34–36] results in low levels of sedimentary and fish tissue total ΣDDTs. Total ΣDDTs in lake sediments and Brook Trout at Anthony and Bennett lakes also likely represent levels typical of atmospheric deposition and weathered inputs in Atlantic Canada [36], including other NB lakes located in watersheds that did not experience direct DDT applications.

Brook Trout in dimictic NB lakes within watersheds where DDT was applied bioaccumulated legacy DDTs, particularly DDDs and DDEs, from the recent sediments to levels greater than Canadian guidelines for wildlife consumers of aquatic biota [8]. Given this and the predictive relationship between lake sediment and legacy DDTs in fish muscle tissue described earlier, lake surface sediments are a good risk predictor of Brook Trout exposure to legacy DDTs in the aquatic food web [24,32]. Pollution from legacy DDTs may now be considered an ecological risk to both aquatic and terrestrial biota that consume most of their prey from NB lake ecosystems. Considering the averages of total ΣDDTs in fish tissue at NB lakes, Brook Trout now exceed the CCME guideline of 14 µg/kg total ΣDDTs for the protection of wildlife consumers of aquatic biota [8] by up to 12.7 times (179 µg/kg, converted wet weight) at California Lake. Additionally, lakes located in watersheds where DDT applications exceeded ~ 0.5 kg/ha may also present risk of Brook Trout muscle tissue surpassing the Canadian guideline for the protection of wildlife consumers of aquatic biota [8]. However, recognizing why lake-specific differences exist between total ΣDDTs in recent sediments and fish tissue is challenging to determine. We speculate that fish growth rate, aquatic food web structure, and conditions governing the breakdown and transport of legacy DDTs in the food web may be factors to consider.

Land use is often associated with organochlorines in fish and aquatic sediments through contaminant inputs via surface water transport [37]. In NB, direct applications of DDT to watersheds by airplanes are also important to consider [6]. However, the relationship between DDTs applied to forests and legacy DDTs measured from environmental storage pools such as sediments and biota is not straightforward. For example, among our impact lakes, Middle Peaked Mountain and Upsalquitch watersheds received comparably high cumulative DDTs. Yet, sedimentary and fish tissue measures of legacy DDTs from these two lakes were not always the highest compared to impact lakes with lower cumulative DDTs applied. Brook Trout from Middle Peaked Mountain showed only the fourth highest total ΣDDTs, whereas its recent sediments total ΣDDTs and its cumulative DDT applied to the watershed at 3.2 kg/ha were the highest of the impact lakes. Kurek et al. [11] showed that Upsalquitch Lake sediments from the 1970s exceeded probable effect levels [7] by about 20, 210, and 450 times for ΣDDT, ΣDDE, and ΣDDD, respectively. However, despite Upsalquitch sediments reflecting extraordinarily high total ΣDDTs in the 1970s at ~ 4500 µg/kg dwt [11], both recent sediments and Brook Trout from Upsalquitch were comparable to other impact lakes with much less DDT applied to the watershed. Additionally, California Lake, with the third highest DDT application rate, showed the highest mean total ΣDDTs in Brook Trout and the second highest in recent sedimentary total ΣDDTs. Differences in DDT applications to each watershed, including the insecticide’s formulation and its pathways into the lake ecosystem are likely important factors to understand lake-specific differences observed.

The preservation and/or breakdown of DDT and its metabolites in the profundal sediments differs between lakes within watersheds of NB where DDT was applied [11], which has implications for bioaccumulation and biomagnification. Our deeper study lakes, California, Upsalquitch, and Middle Peaked Mountain, that range in maximum water depth between ~ 10 and 15 m, tend to show recent sediments with a greater proportion of ΣDDD compared to shallower lakes Goodwin and Sinclair. The relative amounts of the metabolites ΣDDE and ΣDDD in lake sediments provides some evidence that microbial breakdown and environmental conditions in the profundal zone of lakes are important to the signature of legacy DDTs in the aquatic environment [7,35,38,40]. For example, if anoxic/anaerobic conditions at the sediment-water interface commonly occur then bacterial breakdown of DDT typically results in greater relative amounts of DDD, whereas oxygenated conditions tend to favor conversion to DDE [34,38,41]. In late August 2021, dissolved oxygen was measured from each lake’s profundal zone, and all lakes showed values <  1 mg/L, except for Upsalquitch at 2.7 mg/L. However, despite the overall sedimentary signature, total ΣDDTs in Brook Trout muscle tissue from all five impact lakes tend to reflect mostly ΣDDE, which on average comprises ~ 75% of the total ΣDDTs in fish tissue.

The average total ΣDDTs we measured from Brook Trout muscle tissue in five impact lakes ranged from 15 to 179 ng/g wet weight equivalents, thus placing most NB Brook Trout above median values reported from continental-scale surveys of six predatory ( ~ 1 ng/g wet weight) and six bottom-dwelling (12 ng/g wet weight) fish species [42], but at the lower end of exposed Great Lake trout populations assessed three to five decades ago [21]. However, it is challenging to directly compare contaminant results between other North American fish-DDT studies because of differences in fish species, tissues sampled, and/or the lack of recent data. Total ΣDDTs in skin-on Brook Trout muscle tissue from two shallow lakes in northeastern Maine where DDT was once used in military support activities at Loring Air Force Base and in nearby agriculture were studied [43]. Though only a total of four fish were assessed, total ΣDDTs ranged between 141 and 575 ng/g wet weight [43]. Homogenized whole fish total ΣDDTs in 20-30 cm smallmouth bass from a remote lake in the Upper Peninsula of Michigan had a median total ΣDDTs of 340 ng/g wet weight, 84% of which was DDE, on average [22]. In a long-term study, composite whole fish measures of lake trout in Lake Michigan declined between 1970 and 1992 in total ΣDDTs from ~ 19,000 to 1000 ng/g wet weight [21].

### Does food web structure and Brook Trout diet explain legacy DDTs in fish tissue?

Our findings clearly demonstrate that legacy DDTs in lake sediments are now a key pollution pool of ΣDDEs and ΣDDDs, and to a lesser extent ΣDDTs, to the aquatic food web of NB lakes where DDT was applied to the watershed. However, many factors, such as environmental exposure and biological characteristics of organisms, are important to consider with respect to contaminant bioaccumulation in fish [44,45]. For example, Brevik et al. [46] associated sedimentary DDT contamination with DDT levels in fish from a Norwegian lake that received insecticides directly from the sewage outflow of a nearby plant nursery. Seminal research in very large, deep glacial-fed lakes in northern Italy also highlight the importance of sedimentary pools of legacy DDTs to bioaccumulation and its various pathways in the food web [32,33]. Food web structure and availability of prey also influences the bioaccumulation of contaminants, such as DDTs, directly via consumption or indirectly through fish growth rate. Sediment-dwelling prey exposed to high levels of legacy DDTs could be a major vector for bioaccumulation of legacy DDT and its metabolites in aquatic biota through their diet [17], especially in the context of NB lakes due to the extent and magnitude of DDT applied.

We used stable isotopes to determine if food web structure and/or the overall diet of Brook Trout could explain differences of total ΣDDTs in their muscle tissues within and between NB study lakes. As expected, inter-group relative differences in δ^15^N and δ^13^C among consumer and prey were similar across all lakes, reflecting a degree of similarity in productivity regimes, lake size, and nutrient supply. Study-wide similarities in trout δ^15^N and modeling of resource use and trophic position suggest that NB Brook Trout in the sampled size range (i.e., ~ 20-40 cm, 100-500 g) were feeding at a comparable trophic position across all dimictic study lakes [47]. This in turn indicates that variability in Brook Trout diets was not the primary cause of differences in total ΣDDTs in fish among study lakes. However, due to limited sample sizes and our relatively conservative use of 95% credible intervals for comparison, we were unable to reduce model uncertainty to the degree required to resolve the dynamics of trout foraging ecology and DDT accumulation. Overall, our isotope-based findings suggest that Brook Trout diet is a less useful predictor of exposure to legacy DDTs compared to measurement of legacy DDTs in modern lake sediments and/or cumulative DDT applied to the watershed.

## Conclusion

Legacy DDTs, mostly ΣDDE, ΣDDD, and to a lesser extent ΣDDT, are present in sediments and predatory fish in north-central NB lakes at levels that often exceed ecological guidelines. Given both the spatial extent and intensity of DDT applied aerially between 1952 and 1968 in NB, we infer that the chronic effects of legacy DDTs are likely widespread and above levels recognized to harm aquatic biota. Lake sediments now act as a key exposure pool of legacy DDTs to biota in NB watersheds where historical applications of DDTs occurred. Given uncertainties in the rate of breakdown of legacy DDTs and its complex environmental pathways, focused research efforts are needed to determine the relationship between cumulative DDT applications to a watershed and risk to consumers of aquatic biota in lakes, ponds, and wetlands located across more than 50% of the land area of NB.
